# Motivational and emotional determinants of health goal progress in inflammatory bowel disease: a longitudinal study

**DOI:** 10.3389/fpsyt.2026.1864283

**Published:** 2026-09-03

**Authors:** Barbara Horvát, Anett Dávid, Viola Sallay, Beatrix Rafael, Sanela Njers, Kata Orbán, Tamás Molnár, Márta Csabai, Ádám Tóth, Tamás Martos

**Affiliations:** 1 Department of Personality, Clinical and Health Psychology, Institute of Psychology, Faculty of Humanities and Social Sciences, University of Szeged, Szeged, Hungary; 2 Department of Internal Medicine, University of Szeged, Szeged, Hungary; 3 Faculty of Psychotherapy Science, Sigmund Freud Private University, Paris, France; 4 Department of Preventive Medicine, University of Szeged, Szeged, Hungary; 5 Department of Cognitive and Neuropsychology, University of Szeged, Szeged, Hungary; 6 Department of Personality, Clinical and Health Psychology, Szeged, Hungary; 7 Department of Clinical Psychology, Károli Gáspár University of the Reformed Church in Hungary, Budapest, Hungary; 8 Institute of Mechatronics, Faculty of Engineering, Szeged, Hungary

**Keywords:** chronic disease, health behavior, health-related personal goals, inflammatory bowel disease, self-concordance

## Abstract

**Background:**

Self-management among patients with inflammatory bowel disease (IBD) is influenced by motivational and emotional factors. Although research has increasingly highlighted psychological support, it has underexplored interactions among goal-related emotions, motivation types, and progress in health-related behavior.

**Methods:**

A total of 123 adults diagnosed with IBD participated in a longitudinal study, completing questionnaires at baseline (T1) and at follow-ups after three months (T2) and six months (T3). Measures included self-concordance (autonomous and controlled motivation), positive and negative emotions, and goal progress. Longitudinal relations were assessed using a two-step latent-score approach and structural equation modeling.

**Results:**

Autonomous motivation at T1 and T2 predicted more positive emotions at T2 and T3. Positive emotions at T2, in turn, predicted greater autonomous motivation at T3. Controlled motivation at T1 predicted more negative emotions at T2. Additionally, positive emotions at T1 predicted better goal progress at T2, which further increased positive emotions at T3.

**Conclusions:**

The findings identified reciprocal, mutually reinforcing associations between autonomous motivation and positive emotions, as well as between positive emotions and perceived progress toward health-related goals. Supporting these self-reinforcing processes may strengthen the long-term self-regulation required for effective self-management in individuals living with IBD.

## Introduction

### Self-care, self-management, and motivational regulation in IBD

Inflammatory bowel disease (IBD) is a chronic condition of the intestinal tissues that causes abdominal pain, bloody diarrhea, fatigue, and weight loss. Its main clinical forms, Crohn’s disease and ulcerative colitis, alternate between remission and relapse (1). In Hungary, an estimated 50, 000 people are affected by this disease (2, 3).

Recent literature has conceptualized self-care in IBD as a multidimensional construct including behavioral, psychological, and relational domains. Grounded in the Middle-Range Theory of Self-Care of Chronic Illness, self-care in IBD has been described across three interconnected domains: self-care maintenance, self-care monitoring, and self-care management (4, 5). Self-care maintenance refers to routine behaviors to preserve physical and emotional stability, such as medication adherence, dietary regulation, attendance at follow-up visits, and lifestyle behaviors. Self-care monitoring involves the ongoing observation, recognition, and interpretation of disease symptoms or bodily changes. Self-care management refers to the behavioral and cognitive responses to symptoms, when they occur or worsen, such as modifying daily routines or seeking clinical support. Recent findings suggest that patients with IBD generally report adequate levels of self-care maintenance and monitoring, whereas self-care management, particularly symptom interpretation and selecting appropriate responses, remains comparatively more challenging.

Because effective self-care and self-management have been associated with improved health outcomes in patients with IBD, including reduced inflammation, symptom relief, and enhanced quality of life, increasing attention has been given to their psychosocial determinants (6). In particular, psychological resources such as self-efficacy, resilience, and illness perceptions have been shown to predict adherence, symptom control, and quality of life (7, 8). In addition, health-related goals and strivings have been identified as central self-regulatory mechanisms that guide self-management behaviors and facilitate adaptation to chronic illness (9, 10). However, less is known about the motivational and affective processes through which patients regulate their everyday health-related goals. Although previous research has emphasized patient education and behavioral management, the underlying motivational mechanisms supporting self-management remain less clearly understood (11, 12).

### Health-related personal goals

Living with IBD requires ongoing engagement in health-related behaviors such as medication adherence, regular medical follow-up, and lifestyle adjustments (13, 14). However, patients do not pursue these behaviors solely as isolated self-management tasks; they also integrate them into personally meaningful health-related goals. Therefore, in the present study, we used an idiographic goal-assessing approach to examine patients’ health-related personal goals (15, 16).

Health goals are self-identified strivings that patients consider relevant to their health and everyday functioning. Examining these strivings offers insight into patients’ subjective motivational experiences, including how emotionally engaged they are in pursuing health-related goals and how they perceive progress toward them. Progress in these personal pursuits may contribute to a stronger sense of autonomy and self-efficacy; moreover, it may support adaptive engagement in illness-related self-care and self-management, which is closely linked to quality of life in IBD (17, 18). Thus, subjective motivation, emotions, and perceived progress toward health goals may represent motivational-affective processes that support patients’ engagement with self-care and self-management demands in IBD.

### The role of self-concordance in motivational processes

Self-concordance theory (19) posits that goals aligned with an individual’s core values and sense of identity enhance their well-being, motivation, and persistence. This is closely associated with self-determination theory (20, 21) as self-concordant behavior reflects autonomous motivation (AM) rather than actions driven by external pressures or obligations, i.e., controlled motivation (CM).

Emotions are crucial in sustaining health behaviors as they provide feedback on the success of self-management efforts and reinforce habits through reward mechanisms (22). Indeed, the influence of emotional motives on health-related decision-making is often stronger than purely rational considerations (23). Self-concordant goals are particularly enduring because individuals who pursue them feel positive emotions (19, 24, 25). By satisfying basic psychological needs, these goals foster well-being and support long-term commitment to health behaviors (26, 27). Meanwhile, non-self-concordant goals tend to generate less happiness even when they are successfully achieved.

Emerging research has suggested that self-concordance may play a central role in the psychological and health-related functioning of patients with IBD (28, 29). Studies involving IBD patients have confirmed the crucial influence of emotions in psychological functioning, highlighting how patients’ affective experiences interact with goal pursuit and self-management strategies to shape their overall well-being. The current study examines how goal self-concordance may support emotional experiences in pursuing health goals and how positive emotions help sustain perceived progress toward these goals.

### Aims

This study extends prior work by Horvát and colleagues (28–30, which primarily examined connections among motivational and emotional resources (self-concordance, self-efficacy, positive and negative emotions), perceived goal progress, and mental health. Importantly, the present study focuses on perceived progress toward self-identified health-related personal goals, rather than on self-care or self-management behavior per se. Personal goals and perceived progress are conceptually related to self-care and self-management: progress in personally meaningful health goals may support patients’ sense of autonomy, self-efficacy, and adaptive engagement with illness-related demands. Nevertheless, this progress should be distinguished from direct behavioral indicators of self-care maintenance, monitoring, or management.

In sum, the present study aimed to provide a deeper understanding of longitudinal self-regulatory processes in IBD, focusing on the prospective interplay among autonomous and controlled motivation, positive and negative emotions, and perceived progress toward health-related goals, within the broader context of IBD self-care and self-management.

This study grouped the hypothesized mechanisms into two thematic areas, each reflecting a direction of influence within the self-regulation process.

#### Bidirectional dynamics between motivation and emotional experiences

H1: Higher levels of AM at both baseline (T1) and second follow-up (T2) will predict more positive and fewer negative emotions at the subsequent time points (T2 and the third follow-up [T3], respectively).

H2: Higher levels of CM at T1 and T2 will predict greater negative and lower positive emotions at the subsequent time points (T2 and T3, respectively).

H3: Higher levels of positive emotions and lower levels of negative emotions at T1 and T2 will predict greater AM at the subsequent time points (T2 and T3, respectively).

**Figure 1:**
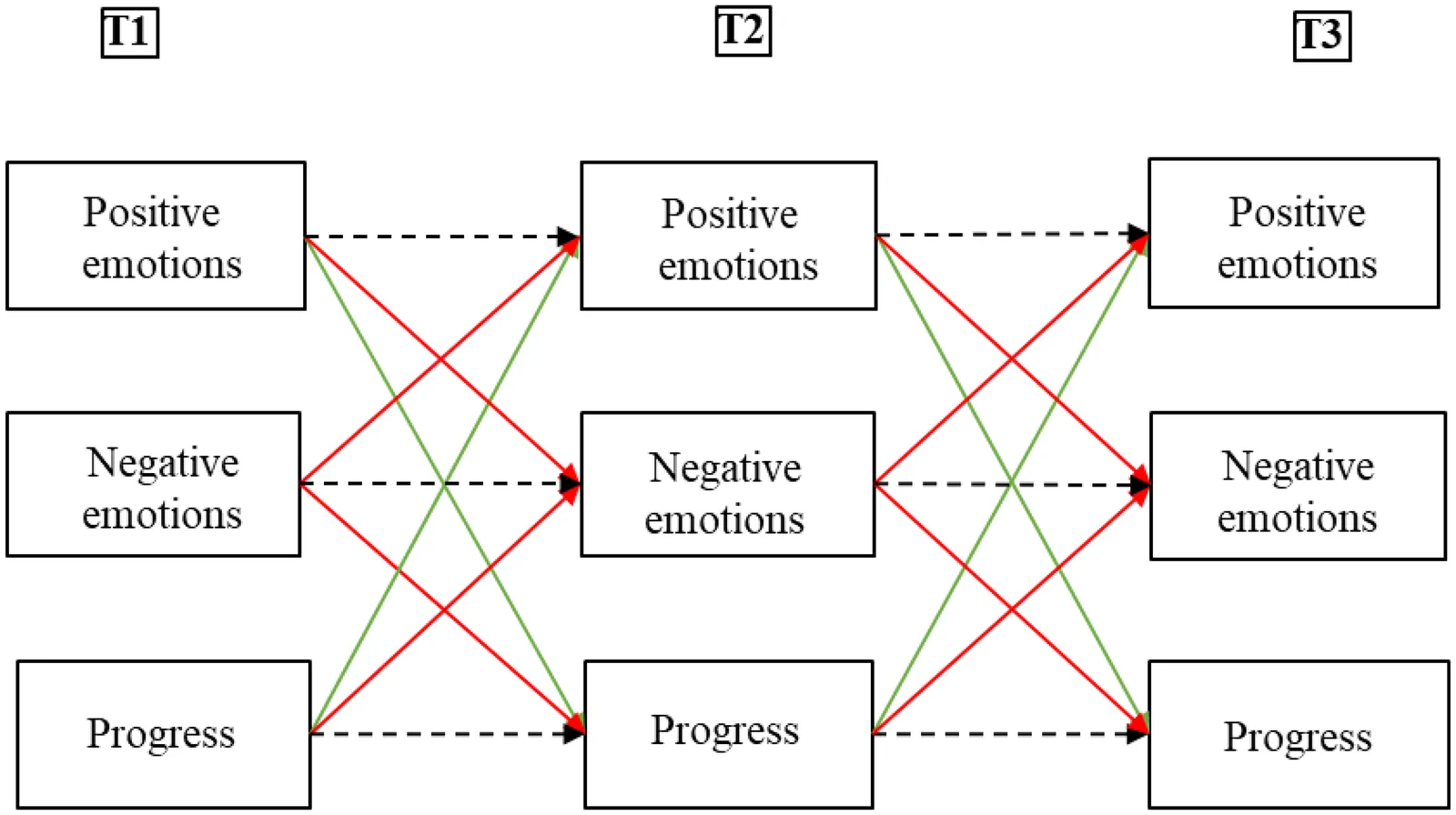
Visual summary of the first model. The statistical analysis is based on covariances. Green lines indicate positive predictions, and red lines indicate negative predictions. Auto-predictions are shown with black dashed lines. Covariances are omitted for clarity.

H4: Higher levels of positive emotions and lower levels of negative emotions at T1 and T2 will predict lower CM at the subsequent time points (T2 and T3, respectively) (**Figure 1**).

#### Bidirectional dynamics between emotions and goal progress

H5: Higher levels of positive emotions and lower levels of negative emotions at T1 will predict greater Pr at T2.

**Figure 2:**
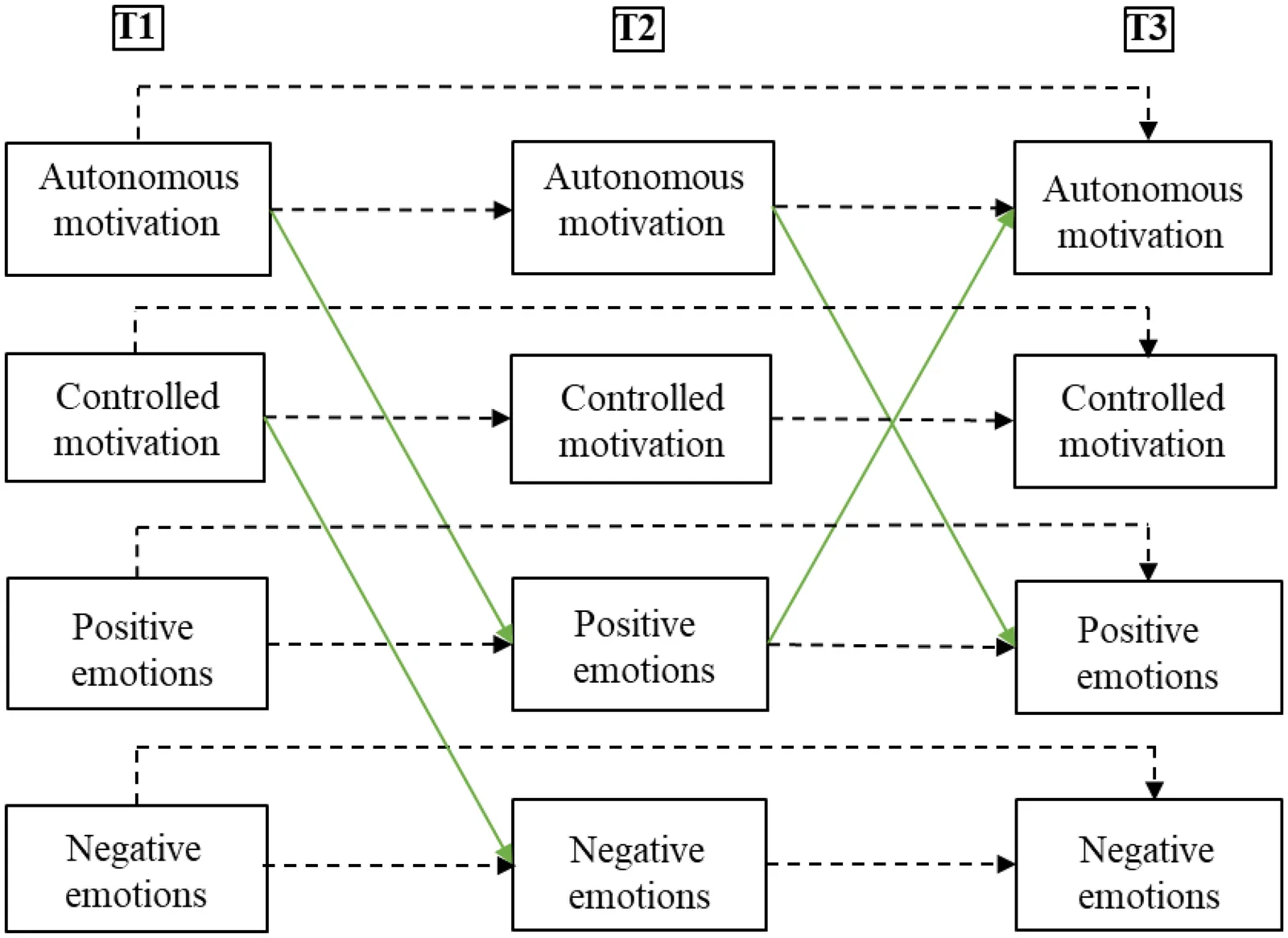
Visual summary of the second model. The statistical analysis is based on covariances. Green lines indicate positive predictions, and red lines indicate negative predictions. Auto-predictions are shown with black dashed lines. Covariances are omitted for clarity.

H6: Greater Pr at T2 will predict higher levels of positive emotions and lower levels of negative emotions at T3 (**Figure 2**).

## Methods

### Study design and procedure

**Figure 3:**
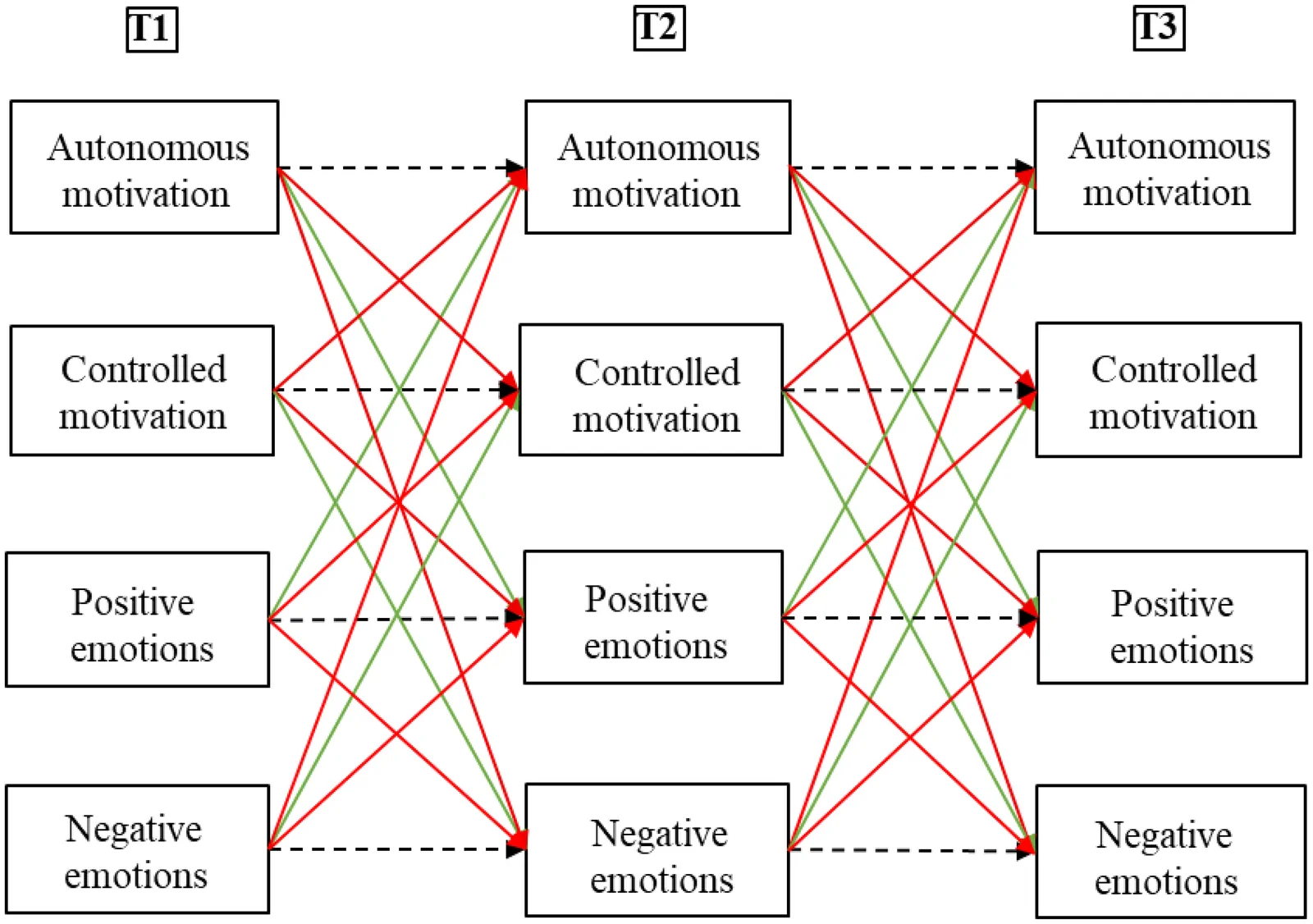
Assessment phases of the study.

This study used a longitudinal, questionnaire-based design. The first baseline (T1) data collection began in November 2022 and continued through February 2023. During this initial recruitment period, 309 participants completed the questionnaire. A second recruitment was conducted in December 2023, using the same methodology, and an additional 88 participants were recruited. We combined data from the two assessments in subsequent analyses. Follow-up assessments were conducted 3 months (T2) and 6 months (T3) after the baseline in both the initial and second samples. Overall, data collection was completed in July 2024. Of the 418 participants recruited at baseline, 191 completed the T2 assessment and 148 the T3 assessment. After screening for missing data and incomplete responses, as described below, a total of 123 participants were included in the current study. **Figure 3** presents an overview of the assessment phases.

Ethical approval was obtained from the Regional Research Ethics Committee (RKEB) of the University of Szeged, Albert Szent-Györgyi Health Centre (approval no. 14/2022-SZTE RKEB). This research was conducted in accordance with the Declaration of Helsinki, and written informed consent was obtained from all participants.

### Participants

The initial data collection (T1) was conducted through in-person recruitment at the Department of Internal Medicine, University of Szeged. Participants were recruited from the clinic’s outpatients receiving ongoing gastroenterological care. Recruitment took place during routine follow-up visits or appointments for biological therapy administration, which typically occurred every 3–4 months. Follow-up assessments at the second (T2) and third (T3) time points were completed online. Participants were included in the study if they (1) were age 18 or older, (2) were diagnosed with IBD according to the international diagnostic criteria, and (3) provided written informed consent. During baseline data collection, participants provided written informed consent and then completed the questionnaire, which took 30–40 minutes. At the end of the baseline questionnaire, after receiving sufficient information about the research design, participants were asked to provide consent to participate in the follow-up assessments. Those who agreed to participate in the T2 and T3 data collection received the corresponding questionnaires by e-mail.

To assess attrition bias, we compared the demographic characteristics of participants at T1 who were included in the final sample (N = 123) with those who dropped out of the longitudinal study (N = 295). We conducted a binomial logistic regression to assess whether age, gender, education, economic status, marital status, disease type, disease status, duration, stenosis, fistula, or prior surgery predicted dropout status (χ²(17) = 44.80, McFadden’s R² = 0.13, Deviance = 295, AIC = 331, p <.001; predictor levels were the same as in **Table 1**). None of the associations reached statistical significance at p <.05, indicating that these characteristics were not robustly associated with non-participation in the follow-up assessments.

**Table 1:** Descriptive characteristics of the study sample.

Socio-demographic characteristic		N (valid percent)
Gender	Male	55 (44.7)
	Female	68 (55.3)
Education	Elementary	1 (.83)
	High school	73 (60.83)
	College or university studies	46 (38.3)
Marital status	Single	22 (18.3)
	In relationship	95 (79.2)
Economic activity	Active (part-time or full-time job)	89 (74.1)
	Inactive	26 (21.6)
	Student	3 (2.5)
Disease type	CD (Crohn’s disease)	87 (72.5)
	UC (Ulcerative colitis)	33 (27.5)
	US (Unspecified)	3 (2.5)
Disease activity (at baseline)	Remission	91 (75.8)
	Relapse	21 (17.5)
Intestinal complication (Stenosis)	Yes	40 (33.3)
	No	54 (45.0)
Intestinal complication (Fistula)	Yes	38 (31.7)
	No	53 (44.2)
SurgerySu Prior surgery	Yes	68 (56.7)
	No	51 (42.5)

Missing responses are not indicated for the categories.

Data were analyzed at three time points (T1, T2, T3). Before the longitudinal analyses, we screened the dataset for response completeness on the manifest variables used in the present study. Respondents were retained if they provided complete or near-complete data across the three waves. Specifically, cases were included if they had complete responses on the relevant manifest variables at all time points, or, in cases with incomplete response patterns, if the proportion of missing entries did not exceed 25% across the variables required for the longitudinal models at any time point. These incomplete but sufficiently informative cases were retained for subsequent FIML-based estimation. In total, 105 complete cases and 18 incomplete but eligible cases were identified across the three time points and included in the longitudinal analyses.

As a result, 123 adults with IBD (55 male, 68 female; mean age: 43.5 years, SD = 11.5) were included in analyses of the baseline (T1), second (T2), and third (T3) follow-up assessments. Of these, 87 were diagnosed with Crohn’s disease, 33 with ulcerative colitis, and 5 with unspecified IBD; the mean disease duration was 18.2 years (SD = 9.5). **Table 1** shows detailed demographic and clinical data for the follow-up sample.

## Materials

Demographic characteristics, including gender, age, socioeconomic status, and marital status, as well as disease-related information such as time since diagnosis, disease type, and symptoms, were collected only at baseline. Psychological characteristics related to health goals were assessed at all three time points. The health goal assessment consisted of the following sections:

### Health-related personal goals

The participants began this part by recalling and describing their personal health-related goals and the activities they had recently engaged in to improve their physical or mental well-being. According to the Personaly Project Analysis method of Little (31), participants begin by **Supplementary Appendix A1** presents examples of these self-identified goals. Subsequently, the relevant constructs were assessed in relation to the participants’ health-related goals as indicated in the T1 assessment. In the T2 and T3 assessments, the questions referred to the same goals as in the T1 assessment.

#### Self-concordance

Self-concordance (19) was assessed as a combination of autonomous and controlled motivation for health-related personal goals, measured separately. These motivation types were measured using two controlled motivation items (CM; e.g., “You are pursuing this goal because you would feel ashamed, guilty or anxious if you didn’t.”) and two autonomous motivation items (AM; e.g., “You pursue this goal because of the fun and enjoyment it provides you.”).

#### Positive and negative emotions

Six items described the participants’ emotional experiences (32) as they pursued their goals. Three of these measured negative emotions (NE; e.g., “How often do you experience negative emotions in your daily life: stress, worry, and anxiety about this goal?”), and three assessed positive emotions (PE; e.g., “How often do you experience positive emotions in your daily life: joy, happiness about this goal?”). Both subscales were rated using a five-point Likert scale.

#### Perceived goal progress

Participants’ perceived progress (Pr) (33, 34) of health-related personal goals was assessed using four items rated on a seven-point Likert scale (1 = very rarely, 7 = very often) (e.g., “I feel that I made good progress in this goal in recent weeks.”).

Internal consistency of the scales was assessed using Cronbach’s alpha coefficients, which indicated acceptable reliability. **Supplementary Appendix A2** shows the means and standard deviations of the questionnaire scores. The correlation matrix of the latent score variables are presented in **Supplementary Appendix 3**.

## Results

### Statistical analyses

We conducted the statistical analyses in JAMOVI (version 2.3; 35). We followed a two-step analytic procedure. As an initial robustness check, we tested whether the two hypothesized longitudinal models could be estimated as fully latent-variable SEMs, with all key constructs derived directly from their manifest indicators across the three waves. These models were not retained because they exhibited convergence problems, most likely due to the high number of freely estimated parameters relative to the available longitudinal sample size. We therefore adopted a two-step latent-score approach to retain a measurement-model-based representation of the constructs while keeping the longitudinal structural models estimable.

In the first step, we estimated longitudinal confirmatory factor analytic models for the study constructs across the three time points and assessed longitudinal measurement invariance. Missing data were handled using full information maximum likelihood (FIML). Based on these measurement models, we saved estimated factor scores for each construct at each wave, yielding 15 latent-score variables corresponding to five constructs measured at three time points. In the second step, we used these estimated factor scores as input variables in cross-lagged panel models specified in line with the study hypotheses. Standardized regression coefficients (β) quantified the strength of association; values around.10,.30, and.50 were interpreted descriptively as small, moderate, and large effects, respectively.

Model fit was evaluated using multiple indices, including the χ² test, the root mean squared error of approximation (RMSEA), the comparative fit index (CFI), and the Tucker–Lewis index (TLI). Following conventional guidelines, CFI and TLI values near or above.95 were considered indicative of good fit, whereas values around.90 were considered acceptable. RMSEA values below.05 were interpreted as good, and those between.05 and.08 as acceptable (36). Because the χ² test is sensitive to sample size and model complexity, it was used primarily to document model fit and compare nested models rather than as the sole criterion for evaluating model adequacy.

### Latent variable estimation and descriptive statistics

Longitudinal measurement models were estimated separately for the five constructs across the three time points. For each construct, configural, metric, and scalar invariance models were tested. Detailed fit indices for each step are documented in **Supplementary Appendix 4**. Overall, the scalar models showed acceptable fit, supporting the use of longitudinally comparable estimated factor scores. Fit was strongest for negative emotions and controlled motivation, whereas the positive emotions and goal progress models showed acceptable but less optimal approximate fit. The two two-indicator motivation models were interpreted with caution because their configural models were just-identified or produced estimation warnings. Estimated factor scores from the scalar models were saved and used in the subsequent cross-lagged panel analyses. **Table 2** shows the descriptive statistics of the latent score variables.

**Table 2:** Descriptive statistics of the latent score variables (N = 123).

Variables	Mean	SD	Minimum	Maximum
AM-T1	.01	.48	-1.37	.75
AM-T2	.01	.35	-1.04	.58
AM-T3	-.01	.51	-1.48	.93
CM-T1	-.01	.35	-0.86	1.23
CM-T2	.01	.14	-0.21	.49
CM-T3	.01	.57	-1.12	1.82
PE-T1	.09	.99	-2.17	1.86
PE-T2	.01	1.01	-2.17	1.89
PE-T3	-.10	.99	-2.26	1.94
NE-T1	.04	.90	-0.78	2.69
NE-T2	-.02	.82	-0.78	2.59
NE-T3	-.02	.82	-0.73	2.62
Pr-T1	.07	.75	-1.63	1.64
Pr-T2	-.02	.66	-1.49	1.45
Pr-T3	-.09	.79	-1.75	1.70

Meaning of abbreviations: AM, autonomous motivation; CM, controlled motivation; PE, positive emotions; NE, negative emotions; Pr, goal progress.

### Path analysis: bidirectional dynamics between motivation and emotions

**Figure 4:**
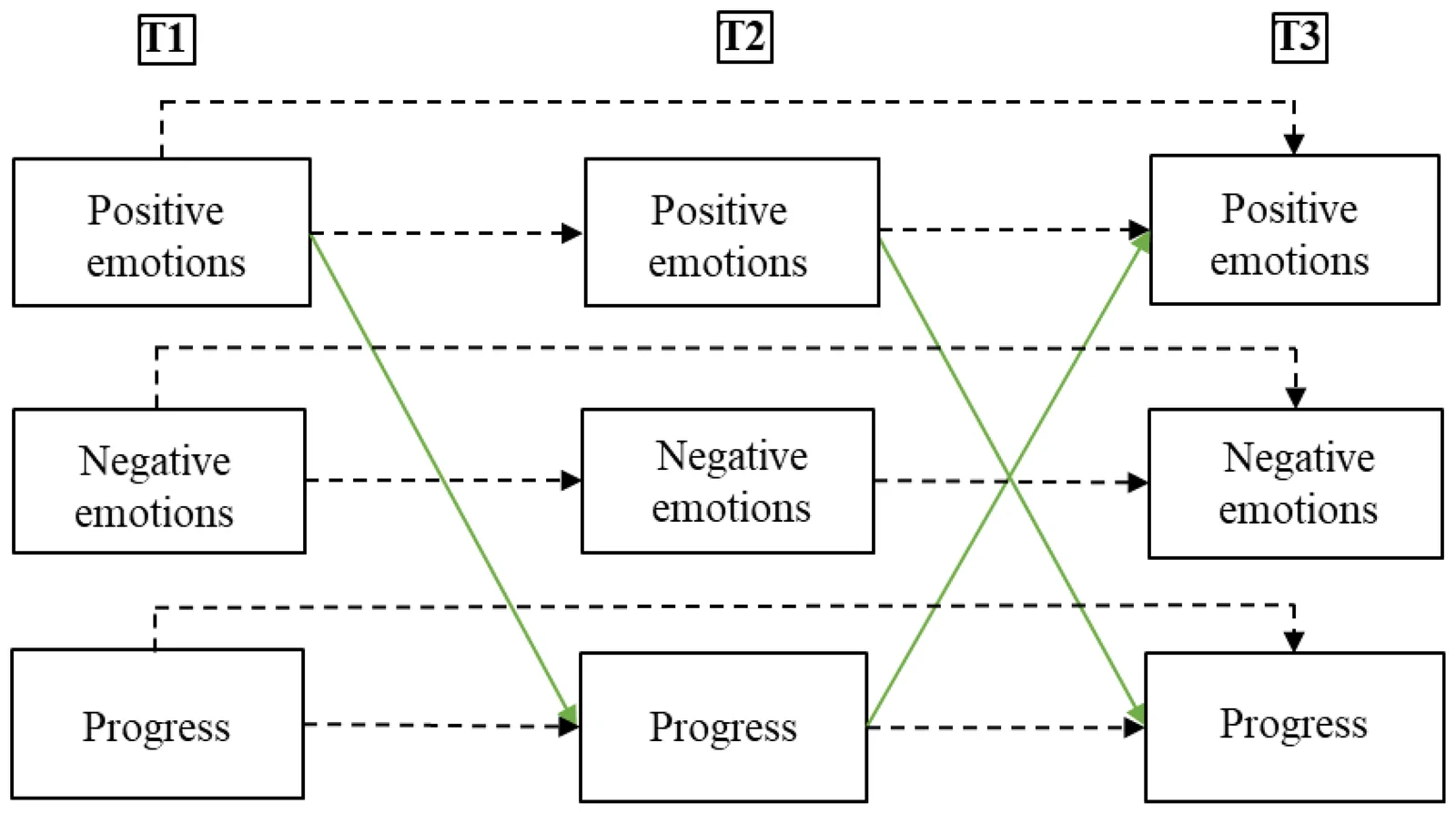
Visual summary of the first model’s significant paths based on covariances. Green lines indicate positive predictions, red lines indicate negative predictions, and black lines indicate autoregressive predictions. Covariances are omitted for clarity.

The hypothetical model in **Figures 1**, **2** was tested using two cross-lagged path models. The first model estimated relationships among positive and negative emotions, AM, and CM across the three waves, T1–T3. Fit indices for this model indicated poor fit to the data: χ2 (16) = 234.0, p < 0.001, CFI = 0.81, TLI = 0.23, RMSEA = 0.33. Inspection of the modification indices, together with the theoretical assumption that baseline levels may exert longer-term stability effects beyond the immediately preceding wave, suggested including direct T1-to-T3 autoregressive paths. We therefore estimated an extended model that included these second-order autoregressive paths. The extended model provided an acceptable fit to the data (χ2 (12) = 19.6, p = 0.076, CFI = 0.99, TLI = 0.96, RMSEA = 0.072), and the cross-lagged paths yielded the same pattern of significant links as the previous model. Consequently, we retained this model as the final specification. **Table 3** shows the final autoregressive and cross-lagged paths for the first model. Results showed that higher AM at T1 and T2 predicted higher PE at T2 and T3, respectively, whereas higher PE at T2 also predicted higher AM at T3. Moreover, higher CM at T1 predicted higher NE at T2. All autoregressive paths, including T1-T3 paths, were significant. **Figure 4** illustrates the significant paths for the FIRST model.

**Table 3:** Autoregressive and cross-lagged paths in the first model.

Paths	Predictor	Dependent	Estimate	SE	*β*	*z*	*p*
T1-T2 auto-regressive paths	PE-T1	PE-T2	.61	.08	.60	7.80	<.001
	NE-T1	NE-T2	.64	.06	.70	9.96	<.001
	AM-T1	AM-T2	.68	.02	.95	31.15	<.001
	CM-T1	CM-T2	.12	.04	.31	3.35	<.001
T1-T2 cross-lagged paths	**AM-T1**	**PE-T2**	**.36**	**.16**	**.17**	**2.24**	**.025**
	AM-T1	NE-T2	-.18	.13	-.11	-1.42	.16
	CM-T1	PE-T2	-.04	.20	-.01	-.21	.83
	CM-T1	NE-T2	-.01	.16	-.00	-.01	.99
	AM-T1	KE-T2	-.02	.03	-.06	-.60	.55
	CM-T1	AM-T2	-.02	.02	-.02	-.78	.43
	PE-T1	AM-T2	.01	.01	.02	.83	.40
	PE-T1	CM-T2	.01	.01	.03	.34	.73
	NE-T1	AM-T2	.01	.01	.01	.19	.85
	NE-T1	CM-T2	.02	.01	.15	1.59	.11
	NE-T1	PE-T2	-0.06	0.08	-0.06	-0.83	.41
	PE-T1	NE-T2	-0.02	0.06	-0.03	-0.35	.73
T2-T3 auto-regressive paths	PE-T2	PE-T3	0.38	0.09	0.39	4.02	<.001
	NE-T2	NE-T3	0.69	0.07	0.69	10.17	<.001
	AM-T2	AM-T3	-0.88	0.34	-0.59	-2.60	.01
	CM-T2	CM-T3	1.02	0.17	0.25	5.82	<.001
T2-T3 cross-lagged paths	**AM-T2**	**PE-T3**	**0.52**	**0.23**	**0.18**	**2.23**	**.03**
	AM-T2	NE-T3	0.15	0.12	0.06	1.17	.24
	CM-T2	PE-T3	-0.36	0.53	-0.05	-0.68	.49
	**CM-T2**	**NE-T3**	**0.70**	**0.29**	**0.12**	**2.42**	**.02**
	AM-T2	CM-T3	0.02	0.07	0.01	0.29	.77
	CM-T2	AM-T3	0.02	0.28	0.01	0.08	.93
	**PE-T2**	**AM-T3**	**0.09**	**0.04**	**0.19**	**2.23**	**.02**
	PE-T2	CM-T3	-0.01	0.02	-0.01	-0.24	.81
	NE-T2	AM-T3	-0.01	0.05	-0.01	-0.17	.87
	NE-T2	CM-T3	-0.05	0.03	-0.07	-1.54	.12
	PE-T2	NE-T3	-0.06	0.04	-0.08	-1.45	.15
	NE-T2	PE-T3	0.16	0.09	0.13	1.71	.09
T1-T3 auto-regressive paths	AM-T1	AM-T3	1.14	0.23	1.07	4.84	<.001
	CM-T1	CM-T3	1.34	0.06	0.81	20.42	<.001
	PE-T1	PE-T3	0.23	0.09	0.22	2.57	.01
	NE-T1	NE-T3	0.14	0.05	0.15	2.57	.01

The values in the table are the standardized coefficients. N = 123.

AM, autonomous motivation; CM, controlled motivation; PE, positive emotions; NE, negative emotions; Pr, goal progress. Significant cross-lagged paths are shown in bold.

### Path analysis: bidirectional dynamics between emotions and progress

The second model examined the relationships between positive and negative goal-related emotions and goal progress across T1–T3. The fit indices of the first model suggested suboptimal data fit: χ^2^ (9) = 52.4, *p* < 0.001, CFI = 0.93, TLI = 0.73, RMSEA = 0.198. Following the same rationale as in the first model, we added direct T1-to-T3 autoregressive paths to account for longer-term stability not captured by the immediately preceding wave. The extended model showed an improved, although still mixed, fit to the data (χ^2^ (6) = 26.9, *p* < 0.001, CFI = 0.97, TLI = 0.80, RMSEA = 0.168).

**Figure 5:**
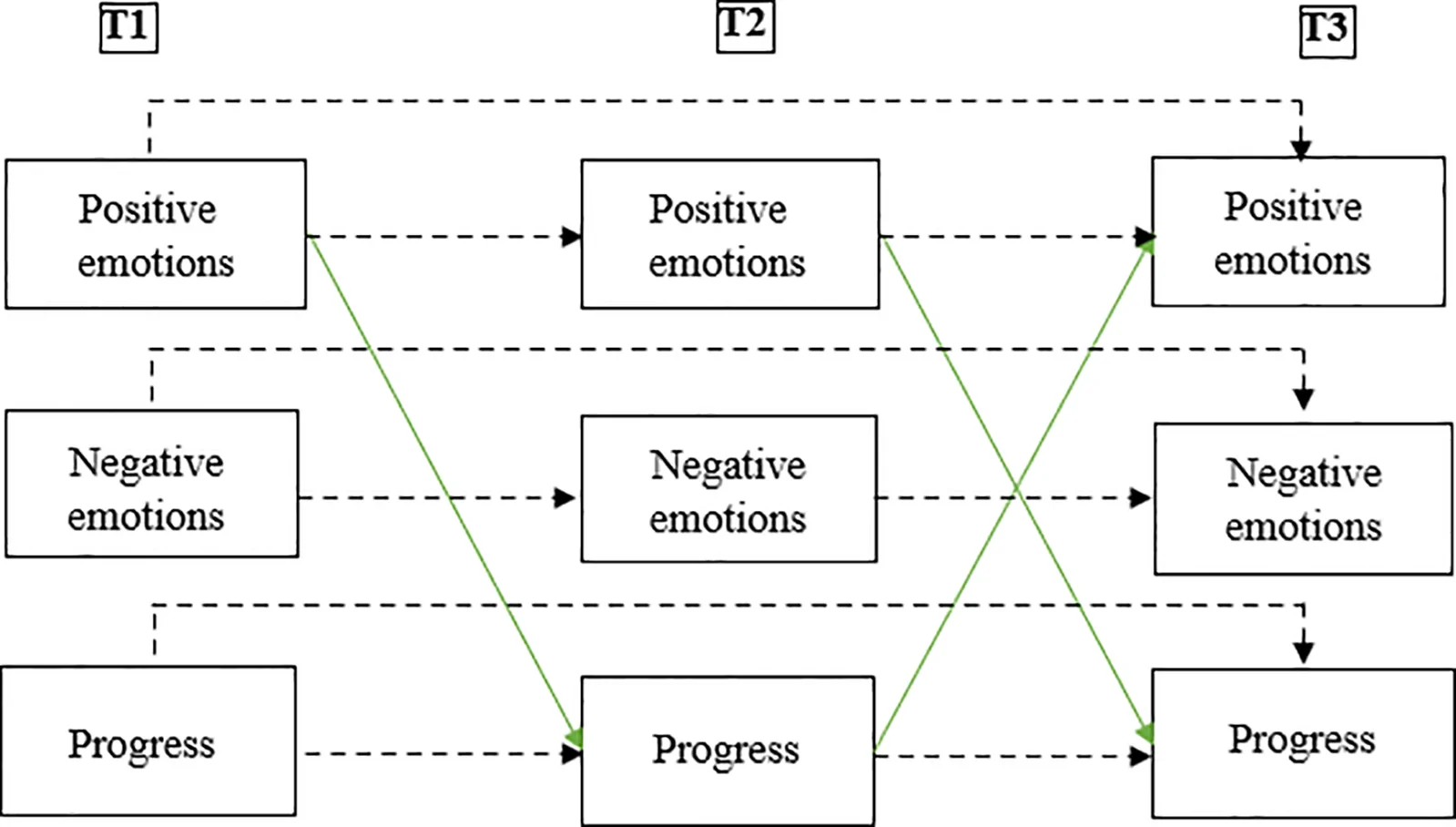
Visual summary of the second model’s significant paths based on covariances. Green lines indicate positive predictions, and black lines indicate autoregressive predictions. Covariances are omitted for clarity.

Importantly, the pattern of significant cross-lagged paths remained unchanged. Because adding additional paths was not theoretically justified, this more parsimonious extended model was retained as the final specification, whereas we acknowledge that its fit remained below conventional thresholds of fit. **Table 4** shows the final autoregressive and cross-lagged paths for the second model. The results indicated that higher PE at T1 predicted higher progress at T2, which in turn predicted higher PE at T3. All autoregressive paths – except for T1-T3 progress – were significant. **Figure 5** illustrates the significant paths for the second model.

**Table 4:** Autoregressive and cross-lagged paths in the second model.

Paths	Predictor	Dependent	Estimate	SE	*β*	*z*	*p*
T1-T2 Auto-regressive paths	PE-T1	PE-T2	.66	.07	.65	9.27	<.001
	NE-T1	NE-T2	.64	.06	.70	11.05	<.001
	Pr-T1	Pr-T2	.36	.07	.41	4.94	<.001
T1-T2 Cross-lagged paths	**PE-T1**	**Pr-T2**	**.14**	**.05**	**.22**	**2.57**	**.01**
	NE-T1	Pr-T2	.08	.06	.12	1.37	.17
	Pr-T1	PE-T2	.14	.09	.10	1.47	.14
	Pr-T1	NE-T2	.01	.07	.01	.18	.86
	PE-T1	NE-T2	-.07	.06	-.09	-1.31	.19
	NE-T1	PE-T2	-.07	.07	-.06	-.95	.34
T2-T3 Auto-regressive paths	PE-T2	PE-T3	.30	.09	.31	3.32	<.001
	NE-T2	PE-T3	.10	.08	.09	1.27	.20
	Pr-T2	Pr-T3	.89	.08	.75	10.92	<.001
T2-T3 Cross-lagged paths	PE-T2	Pr-T3	.01	.05	.02	.29	.78
	NE-T2	Pr-T3	.03	.06	.03	.47	.64
	**Pr-T2**	**PE-T3**	**.22**	**.11**	**.14**	**1.99**	**.05**
	Pr-T2	NE-T3	-.07	.06	-.05	-1.12	.26
	PE-T2	NE-T3	-.01	.04	-.00	-.08	.94
	NE-T2	NE-T3	.72	.07	.71	10.66	<.001
T1-T3 auto-regressive paths	PE-T1	PE-T3	.37	.08	.37	4.42	<.001
	NE-T1	NE-T3	.17	.06	.18	2.78	.005
	Pr-T1	Pr-T3	.02	.06	.02	.30	.76

The values in the table are the standardized coefficients. N = 123.

PE, positive emotions; NE, negative emotions; Pr, goal progress. Significant cross-lagged paths are shown in bold.

## Discussion

Using a longitudinal design, this study examined the bidirectional relationship between self-concordance and emotions in health-related personal goals and explored the predictive role of emotional experiences in goal progress. AM was expected to lead to more positive and fewer negative emotions at later time points, whereas CM was expected to do the opposite. We further hypothesized that positive emotions predict higher AM and lower CM over time. We then assumed that positive emotions predict greater progress, which would enhance positive emotions and reduce negative ones. Although not all hypothesized associations were supported, the significant paths revealed a coherent pattern linking motivation and emotional experiences. AM predicted more positive emotions, whereas CM predicted more negative emotions. In turn, positive emotions predicted subsequent autonomous motivation, suggesting a potentially self-reinforcing cycle between autonomous forms of regulation and beneficial affective experiences. The findings also indicated that positive emotions facilitated goal advancement, and that experiencing progress was associated with further increases in positive emotions. Together, these results point to dynamic interactions among motivation, affect, and self-regulatory processes during goal pursuit.

The finding that higher autonomous motivation at T1 predicted more positive emotions at T2, and that autonomous motivation at T2 predicted more positive emotions at T3, suggests that autonomous motivation fosters sustained positive emotional experiences over time. By contrast, higher controlled motivation (CM) at T1 predicted more negative emotions at T2. These results are consistent with the self-concordance model of goal setting proposed by Sheldon and Elliot (19). This model posits that goals pursued for autonomous reasons are more likely to generate positive affective experiences over time because they reflect individuals’ authentic interests and values, whereas controlled motives are less conducive to psychological well-being.

The present findings align with recent evidence that self-concordant (i.e., autonomously motivated) goals promote more adaptive, engagement-oriented emotion regulation over time (37). They further extend this literature by demonstrating distinct longitudinal associations between autonomous motivation (AM) and controlled motivation (CM) and emotional experiences. By distinguishing between AM and CM, rather than treating motivation as a single dimension, the present study offers a more precise understanding of how self-concordance supports effective self-management among individuals with IBD (28–30). Specifically, this distinction helps identify the motivational processes most likely to sustain engagement in health-related behaviors and to facilitate more adaptive emotional responses to the ongoing challenges of living with a chronic illness.

The present findings suggest that autonomous motivation may contribute to sustained positive emotional experiences, which, in turn, could support long-term self-regulation. This may be particularly relevant for individuals with IBD, who face ongoing demands related to symptom monitoring, treatment adherence, and lifestyle management. Because the disease is characterized by an unpredictable course with recurrent periods of relapse and remission, patients are required to continuously adapt their self-management strategies while coping with both physical symptoms and emotional distress. Sustaining self-regulatory capacity may therefore be essential for maintaining consistent health-related behaviors and effectively managing the challenges associated with living with IBD (38).

Our analysis of the potential feedback function of emotions within the self-concordance framework extends previous research, which has primarily focused on how motivation shapes emotional experiences. The present findings suggest that emotions may also shape subsequent motivational regulation. Specifically, positive emotions at T2 predicted higher autonomous motivation (AM) at T3, consistent with self-determination theory (21), which holds that positive affective experiences signal the satisfaction of basic psychological needs and facilitate the internalization and maintenance of autonomous motivation. In this way, positive emotions may support effective self-management by reinforcing self-endorsed, intrinsically valued behaviors.

A study of educational settings found that higher levels of positive experiences and subjective well-being were associated with greater AM and reduced CM over time. Longitudinal findings by Bieg and colleagues (39) showed that positive affect supports intrinsic motivation (AM), reducing reliance on controlled regulation in learning contexts. The current results extend this evidence to the IBD context, showing that even in a chronic health setting, positive emotions can increase AM by satisfying psychological needs and promoting more autonomous regulation, while negative emotions can foster CM. This emphasizes that motivational quality is dynamically influenced by emotional feedback, not solely by prior goal pursuit.

Hypotheses H3 and H4 examined the longitudinal interplay between positive emotions and perceived progress (Pr). Among individuals with IBD pursuing health-related personal goals, the findings suggest that both experiencing progress and deriving emotional reward from everyday self-management activities may contribute to sustained engagement in health behaviors and strengthen self-determination over time. Specifically, higher levels of positive emotions at T1 and T2 predicted greater perceived progress at T2 and T3, respectively, while perceived progress at T2 predicted higher levels of positive emotions at T3. Together, these findings point to a reciprocal and reinforcing relationship between positive emotions and goal progress, in which positive affect facilitates goal advancement and experiences of progress subsequently enhance positive emotional experiences. This upward cycle may help sustain motivation and engagement in self-management behaviors over time.

Individuals who feel a sense of accomplishment and competence as they move toward their goals (40) find that their basic psychological needs for competence, autonomy, and relatedness are met through the achievement of personally meaningful objectives. Accomplishing these goals yields intrinsic rewards, generating positive emotions such as joy and satisfaction. These emotions, in turn, strengthen their commitment to continued goal pursuit. This is also consistent with Fredrickson’s (41) broaden-and-build theory, which holds that positive emotions such as joy, enthusiasm, and hope expand people’s thinking and behavioral options. They also increase creativity, openness, and persistence, making it easier to achieve these goals. Individuals who experience positive emotions more frequently tend to exert more effort and maintain higher motivation, sustaining progress over time.

Finally, there are a few methodological lessons we can reflect on. First, to our knowledge, this is the first longitudinal study to examine health-related personal goals among individuals with IBD, using an idiographic goal-assessing approach to investigate the motivational and emotional dynamics of these goals over time in relation to goal attainment.

Second, the current results suggest that future self-concordance-based studies may benefit from treating AM and CM as separate constructs rather than using the AM–CM composite index. Drawing on our experience shortening the questionnaire for the longitudinal study, brief scales may serve as useful research proxies; however, in the context of psychological consultation or clinical assessment, a more comprehensive evaluation may provide additional valuable information.

For the statistical analyses, we included direct autoregressive paths from T1 to T3 to improve model fit. Although this modification was initially suggested by inspection of the modification indices, we considered it theoretically justifiable. Specifically, direct predictions from baseline to the third measurement may reflect longer-term stability beyond autoregressive effects, indicating that individuals’ initial motivational and emotional experiences continue to influence their functioning over time. If replicated in future studies, these findings would suggest that baseline interventions aimed at fostering more autonomous and less controlled motivation may have enduring benefits for motivational and emotional functioning.

Finally, it is also important to note that several hypothesized cross-lagged pathways were not supported. Negative emotions did not consistently predict lower autonomous motivation, higher controlled motivation, or lower goal progress over time, and controlled motivation showed only limited prospective associations with later emotional experiences. Thus, the longitudinal pattern was more specific than originally expected: the most robust prospective associations involved autonomous motivation, positive emotions, and perceived goal progress. In addition, some autoregressive estimates, particularly in models including direct T1-to-T3 paths, should be interpreted primarily as statistical controls for temporal stability rather than as substantive indicators of psychological change. This caution is especially relevant because the inclusion of second-order autoregressive paths may introduce suppression-like patterns among adjacent autoregressive coefficients.

## Limitations

The results of the study must be evaluated in light of several limitations. A primary limitation of this longitudinal study was the relatively high attrition rate during the follow-up period. To minimize participant dropout, detailed information about the study was provided during the baseline (T1) assessment, and multiple reminders were sent before each follow-up assessment. Moreover, we did not identify robust individual predictors of attrition. Nevertheless, attrition may have affected the interpretation and generalizability of the findings, as the results primarily reflect the responses of participants who remained engaged and completed all three waves of data collection. This challenge may be partly attributable to the frequent involvement of patients attending the Gastroenterology Ambulance of the Internal Medicine Clinic of the University of Szeged in research activities, which could have reduced their motivation to participate in an additional longitudinal study. Second, the lack of a control group without IBD limits our ability to determine whether the observed associations are specific to IBD patients. Third, disease activity was assessed using self-reported measures rather than validated clinical indices, which may affect the accuracy of the data. In addition, self-management was not measured directly but inferred through proxy variables such as goal progress and emotional states, which may limit the precision of the findings. Future research should complement subjective self-reports with validated clinical indices to enhance measurement accuracy. In addition, self-management should be assessed more directly, for example, through self-monitoring tools within intensive longitudinal designs, allowing for a more precise and comprehensive evaluation of this construct.

## Conclusions and clinical implications

From a clinical perspective, the observed longitudinal associations between motivation quality and emotional experiences highlight the dynamic nature of self-regulation in individuals with IBD. Autonomous motivation predicted subsequent positive emotions, whereas controlled motivation predicted subsequent negative emotions. In turn, positive emotions fostered autonomous motivation and were associated with greater perceived progress toward health-related goals, which in turn increased positive emotions. These findings suggest that individuals who pursue health-related goals for autonomous reasons are more likely to experience goal pursuit as satisfying and emotionally rewarding, which may facilitate further internalization of these goals. Positive emotional experiences may, in turn, help sustain engagement in health-related goal pursuit, while perceiving progress may further reinforce positive emotions. Together, these findings point to a reciprocal, self-reinforcing interplay among autonomous motivation, emotional experiences, and perceived progress.

The present findings offer valuable insight into how long-term self-regulation may be supported in individuals living with IBD. Specifically, they suggest that interventions that foster autonomous motivation and promote positive emotional experiences during health-related goal pursuit may initiate a self-reinforcing process in which motivation, positive emotions, and perceived goal progress strengthen one another over time. Such an upward cycle may enhance patients’ capacity to sustain engagement in health-related behaviors, including treatment adherence, symptom monitoring, and lifestyle management, despite the ongoing challenges associated with the relapsing-remitting nature of IBD.

## Data Availability

The original contributions presented in the study are included in the article/**Supplementary Material**. Further inquiries can be directed to the corresponding author.
