# Supplementary material for: Motivational and emotional determinants of health goal progress in inflammatory bowel disease: a longitudinal study
Source: Front Psychiatry. 2026 Sep 3;17:1864283. doi: 10.3389/fpsyt.2026.1864283 (PMC13584826; doi:10.3389/fpsyt.2026.1864283)
Supplement: Supplementary file 1 [file Table1.pdf]

Supplementary materials

Appendix 1. Examples from the participants' health-related personal goals

Category	Examples
**Diet and healthy eating**	• *Balanced diet*<br>• *Eating healthy*<br>• *Following a diet*
**Meditation**	• *I meditate*<br>• *Contemplative retreat (30 min/day)*<br>• *Meditation for mental health*
**Medical Consultation**	• *Consulting with a doctor*<br>• *Health counseling*<br>• *Bone density test*
**Psychological Health**	• *Visiting a psychologist*<br>• *Relaxation*<br>• *Avoiding stress*
**Spirituality**	• *Studying the Bible*<br>• *Meditation*<br>• *Relaxation*
**Physical activity**	• *Working out*<br>• *Cycling*<br>• *Starting sports*
