# Supplementary material for: Motivational and emotional determinants of health goal progress in inflammatory bowel disease: a longitudinal study
Source: Front Psychiatry. 2026 Sep 3;17:1864283. doi: 10.3389/fpsyt.2026.1864283 (PMC13584826; doi:10.3389/fpsyt.2026.1864283)
Supplement: Supplementary file 2 [file Table2.pdf]

Appendix 2. Means and standard deviations of the observed variables (N = 124)

**Data collection point**	**Variable name**	***M***	***SD***
**T1**	Autonomous motivation	3.73	0.10
	Controlled motivation	1.91	0.94
	Positive emotions	3.29	0.92
	Negative emotions	1.92	1.10
	Goal progress	3.06	0.90
**T2**	Autonomous motivation	3.70	0.84
	Controlled motivation	2.02	0.87
	Positive emotions	3.15	0.97
	Negative emotions	1.82	1.02
	Goal progress	2.94	0.82
**T3**	Autonomous motivation	3.65	0.97
	Controlled motivation	2.02	0.99
	Positive emotions	3.03	0.98
	Negative emotions	1.83	0.99
	Goal progress	2.84	0.94
