# Supplementary material for: Motivational and emotional determinants of health goal progress in inflammatory bowel disease: a longitudinal study
Source: Front Psychiatry. 2026 Sep 3;17:1864283. doi: 10.3389/fpsyt.2026.1864283 (PMC13584826; doi:10.3389/fpsyt.2026.1864283)
Supplement: Supplementary file 3 [file Table3.pdf]

Appendix 3. Longitudinal measurement invariance models for the five constructs

Models were estimated separately for each construct across the three time points.

Construct	Model	χ²	df	p	CFI	TLI	RMSEA [95% CI]	ΔCFI	ΔRMSEA	Constraint score p	Notes
AM	Configural	0.663	0		.997	1.000	.000 [.000, .000]				Two-indicator model; configural model df = 0; estimation warning in output.
AM	Metric	5.27	2	.072	.985	.890	.116 [.000, .241]	-.012	.116	.116	
AM	Scalar	9.99	6	.125	.982	.955	.074 [.000, .152]	-.003	-.042	.188	Factor scores saved from this model.
CM	Configural	0.065	0		1.000	1.000	.000 [.000, .000]				Two-indicator model; configural model df = 0; estimation warnings in output.
CM	Metric	2.19	2	.334	.999	.990	.028 [.000, .184]	-.001	.028	.351	
CM	Scalar	3.95	6	.684	1.000	1.037	.000 [.000, .091]	.001	-.028	.695	Factor scores saved from this model.
PE	Configural	32.8	15	.005	.973	.935	.098 [.052, .144]				
PE	Metric	45.2	19	< .001	.960	.925	.106 [.066, .146]	-.013	.008	.020	Approximate invariance; constraint score test significant.
PE	Scalar	52.9	25	< .001	.958	.939	.095 [.059, .131]	-.002	-.011	.040	Approximate scalar fit; factor scores saved from this model.
NE	Configural	14.0	15	.522	1.000	1.003	.000 [.000, .080]				
NE	Metric	14.9	19	.726	1.000	1.009	.000 [.000, .059]	.000	.000	.928	
NE	Scalar	19.0	25	.797	1.000	1.011	.000 [.000, .048]	.000	.000	.895	Factor scores saved from this model.
Pr	Configural	89.9	39	< .001	.953	.921	.103 [.075, .131]				Approximate fit.
Pr	Metric	92.1	45	< .001	.957	.937	.092 [.065, .119]	.004	-.011	.903	Approximate fit.
Pr	Scalar	105	51	< .001	.950	.935	.093 [.068, .118]	-.007	.001	.225	Approximate scalar fit; factor scores saved from this model.

Note. AM = autonomous motivation; CM = controlled motivation; PE = positive emotions; NE = negative emotions; Pr = perceived goal progress. All models included same-item residual covariances across waves. ΔCFI and ΔRMSEA are calculated relative to the previous invariance level. Constraint score p refers to the total score test for the equality constraints in the metric or scalar model. Empty p values for configural models with df = 0 indicate just-identified models. Factor scores used in the longitudinal analyses were saved from the scalar models.
