# Supplementary material for: Motivational and emotional determinants of health goal progress in inflammatory bowel disease: a longitudinal study
Source: Front Psychiatry. 2026 Sep 3;17:1864283. doi: 10.3389/fpsyt.2026.1864283 (PMC13584826; doi:10.3389/fpsyt.2026.1864283)
Supplement: Supplementary file 4 [file Table4.pdf]

Appendix 4. Correlation matrix of the latent score variables (N =123)

		AM-T1	AM-T2	AM-T3	KM-T1	KM-T2	KM-T3	PE-T1	PE-T2	PE-T3	NE-T1	NE-T2	NE-T3	Pr-T1	Pr-T2	Pr-T3
AM-T1	r	—														
	p	—														
AM-T2	r	.96	—													
	p	<.01	—													
AM-T3	r	.60	.53	—												
	p	<.001	<.001	—												
KM-T1	r	.08	.06	.10	—											
	p	.35	.49	.27	—											
KM-T2	r	-0.03	-0.04	0.01	0.37	—										
	p	.76	.70	.99	<.01	—										
KM-T3	r	.09	.06	.12	.88	.53	—									
	p	.35	.47	.18	<.001	<.001	—									
PE-T1	r	.56	.55	.49	.05	-.01	.01	—								
	p	<.001	<.001	<.001	.56	.95	.69	—								
PE-T2	r	.51	.53	.42	.01	.05	.03	.70	—							
	p	<.001	<.001	<.001	.95	.62	.73	<.001	—							
PE-T3	r	.45	.48	.60	.18	.01	.12	.62	.60	—						
	p	<.001	<.001	<.001	.04	.88	.24	<.001	<.001	—						
NE-T1	r	-.10	-.10	-.01	.43	.28	.37	-.16	-.18	.08	—					
	p	.29	.27	.92	<.001	.002	<.001	.08	.05	.39	—					
NE-T2	r	-.19	-.23	-.13	.29	.41	.27	-.20	-.28	-.08	.71	—				
	p	.04	.01	.16	.001	<.001	.01	.03	.01	.35	<.001	—				

		AM-T1	AM-T2	AM-T3	KM-T1	KM-T2	KM-T3	PE-T1	PE-T2	PE-T3	NE-T1	NE-T2	NE-T3	Pr-T1	Pr-T2	Pr-T3
NE-T3	r	-.13	-.16	-.22	.29	.44	.30	-.24	-.26	-.18	.66	.86	—			
	p	.16	.08	.02	.01	<.001	<.001	.01	.01	.05	<.001	<.001	—			
Pr-T1	r	.28	.31	.25	.01	.09	-.02	.41	.37	.35	-.12	-.12	-.06	—		
	p	.01	<.001	.01	.92	.29	.86	<.001	<.001	<.001	.19	.24	.49	—		
Pr-T2	r	.13	.19	.12	.08	-.06	-.02	.36	.40	.39	.02	-.08	-.11	.49	—	
	p	.14	.04	.18	.37	.52	.85	<.001	<.001	<.001	.80	.39	.23	<.001	—	
Pr-T3	r	.13	.15	.23	.14	-.12	.01	.26	.31	.51	.19	-.04	-.12	.38	.76	—
	p	.16	.09	<.001	.11	.21	.98	.003	<.001	<.001	.03	.68	.19	<.001	<.001	—
